# Psychophysiological stress-reactivity in clinical and non-clinical voice-hearers

**DOI:** 10.1016/j.schres.2021.07.005

**Published:** 2021-09

**Authors:** David Baumeister, Toby Pillinger, Oliver Howes, Emmanuelle Peters

**Affiliations:** a Institute of Psychiatry, Psychology & Neuroscience, King's College London, Department of Psychology, London, UK; b Department of General Internal Medicine and Psychosomatics, University Hospital Heidelberg, Germany; c Institute of Psychiatry, Psychology & Neuroscience, King's College London, Department of Psychosis Studies, London, UK; d South London and Maudsley NHS Foundation Trust, London, UK

**Keywords:** Stress, Cortisol, HPA Axis, Psychosis continuum, Hallucinations, Voice-hearing

## Abstract

**Background:**

Psychosis is associated with dysregulation of psychophysiological stress-reactivity, including in subjective, autonomic nervous system (ANS) and hypothalamic-pituitary-adrenal (HPA) parameters.

**Aims:**

This study investigated whether dysregulated psychophysiological stress-reactivity is specifically associated with auditory verbal hallucinations (AVHs) or psychosis more generally by comparing voice-hearers with and without a need for care.

**Method:**

Clinical (*n* = 20) and non-clinical voice-hearers (*n* = 23), as well as a healthy control group with no voices (n = 23), were compared on HPA and ANS responses, and subjective reactivity, to a psychophysiological stress paradigm, the socially evaluative cold pressor test.

**Results:**

Measures of HPA function in both clinical and non-clinical voice-hearers diverged from non-voice-hearing controls. Clinical participants showed a blunted peak response compared to both non-clinical groups (*p* = 0.02), whilst non-clinical voice-hearers showed, at trend-level, reduced cortisol levels during stress exposure compared to both clinical voice-hearers (*p* = 0.07) and healthy controls (p = 0.07), who unexpectedly did not differ from each other (*p* = 0.97). Clinical participants showed greater subjective stress levels than both non-clinical groups (*p* < 0.001), as well as greater anticipatory stress (*p* = 0.001) and less recovery. There were no differences between groups on parameters of the ANS (all *p* > 0.05).

**Conclusions:**

Dysregulated psychophysiological stress-function is present in clinical voice-hearers, and partially discriminates them from non-clinical voice-hearers. Overall, the present findings identified specific potential psychophysiological markers of risk and resilience in auditory verbal hallucinations and need for care.

## 1 Introduction

Diathesis-stress models propose that the interaction of genetic vulnerability and stress exposure leads to aberrant stress-function and the emergence of psychosis (Howes et al., 2017; Howes and Murray, 2014; Pruessner et al., 2017). Psychosis patients are more likely to appraise events in daily life as stressful (Myin-Germeys et al., 2001), daily life stressors are positively associated with intensity of psychotic experiences (Myin-Germeys et al., 2005) and elevated stress sensitivity to daily events as well as increased threat anticipation are associated with greater rates of psychotic experiences (Reininghaus et al., 2016). Physiologically, the sympathetic branch of the autonomic nervous system (ANS) is unaltered in psychosis, whereas activity of the parasympathetic branch is dampened, meaning that there is an imbalance in ANS activity (Montaquila et al., 2015). Two studies have reported increased levels of salivary α-amylase in psychosis, a putative biomarker of ANS balance (Ieda et al., 2014; Inagaki et al., 2010). Further, there is evidence for altered hypothalamic-pituitary-adrenal (HPA) function in psychosis, with evidence of a blunted response to psychosocial stress (Ciufolini et al., 2014). However, psychotic disorders are highly heterogeneous, and a link between stress-reactivity and specific symptoms has not been investigated.

Recent research has investigated psychotic experiences and auditory verbal hallucinations in people without a diagnosis of a psychotic disorder and who are otherwise healthy (Baumeister et al., 2017; Johns et al., 2014; Peters et al., 2016). The current literature suggests that AVHs in non-clinical voice hearers (NCVH) are very similar to those of clinical voice-hearers (CVH) in terms of acoustic phenomenology, but do not have the same distressing content or impact (Baumeister et al., 2017). The ability of an individual to respond to stress in an adaptive manner and overcome stress exposure may be a key variable in determining need for care. In view of this we hypothesised that clinical voice hearers would show an abnormal HPA axis relative to non-clinical voice hearers, who would show responses similar to controls. To address this question, the present study set out to compare physiological stress-reactivity in CVHs and NCVHs, and healthy controls with no AVHs (HCs).

Several experimental stress parameters were measured to allow for a comprehensive assessment of stress-reactivity. To investigate acute reactivity of the HPA axis and the ANS to a psychobiological stress induction, the socially-evaluative cold pressor test (SECPT; Schwabe et al., 2008) with salivary assessment of cortisol and α-amylase was chosen, as well as subjective reactivity to the stressor and anticipatory stress appraisal.

CVHs, compared to the other two groups, were hypothesised to:

a) show an elevated salivary α-amylase reaction in the context of a blunted cortisol response to the SECPT and higher overall cortisol levels;
b) show increased anticipatory stress appraisal before exposure and have higher subjective stress levels throughout the paradigm, including greater overall levels and diminished recovery.

On all parameters, NCVHs were hypothesised not to differ from HCs.

## 2 Methods

### 2.1 Sample

The authors assert that all procedures contributing to this work comply with the ethical standards of the relevant national and institutional committees on human experimentation and with the Helsinki Declaration of 1975, as revised in 2008. Ethical approval was granted by the NRES Committee London Dulwich (15/LO/0880). All participants provided informed consent. Twenty CVHs were recruited from outpatient services of the South London and Maudsley NHS Foundation Trust. Twenty-three NCVHs were recruited from community samples and specialist organisations in line with our group's previous strategy (Peters et al., 2016). Finally, 23 HCs were recruited by opportunity sampling from the general population. Sample characteristics are presented in Table 1.

**Table 1:** Sample descriptives (mean ± SD, [% exposed]).

	CVHs (n = 20)	HVHs (*n* = 23)	HCs (n = 23)	Statistics	CVH vs HVH	CVH vs HC	HVH vs HC
Age	47.7 ± 8.5	45.24 ± 14.4	*45.2* ± 10.4	*F* (65) = 0.3, *p* = 0.7, d = 0.2, BF_10_ = 0.1	*–*	*–*	
Gender (% female)	35.0%	64.0%	*30.4%*	***χ***^***2***^ ***= 6.5, p = 0.04,*** ***v = 0.31, BF***_***10***_ ***= 2.4***	*χ*^*2*^ = 3.7, *p* = 0.05, d = 0.60, BF_10_ = 2.2	*χ*^*2*^ = 0.1, *p* = 0.75, d = 0.10, BF_10_ = 0.4	***χ***^***2***^ ***= 5.4, p = 0.02,*** ***d = 0.71***, **BF**_**10**_ **= 4.9**
Ethnicity (% White British)	65.0%	52.0%	*60.9%*	*χ*^*2*^ = 0.8, *p* = 0.7, v = 0.11, BF_10_ = 0.2	*–*	*–*	*–*
First language (% English)	75.0%	80.0%	*87.0%*	*χ*^*2*^ = 1.0, *p* = 0.6, v = 0.12, BF_10_ = 0.1	*–*	*–*	*–*
Employment (% unemployed)	100.0%	16%	*8.7%*	***χ***^***2***^ ***= 46.0, p < 0.001,*** ***v = 0.82, BF***_***10***_ ***= 7.2 × 10***^***9***^	***χ***^***2***^ ***= 31.5, p < 0.001,*** ***d = 3.06***, **BF**_**10**_ **= 2.5 × 10**^**7**^	***χ***^***2***^ ***= 35.7, p < 0.001,*** ***d = 4.42***, **BF**_**10**_ **= 3.6 × 10**^**8**^	*χ*^*2*^ = 0.6, *p* = 0.4, d = 0.23, BF_10_ = 0.3
Living status (% alone)	57.1%	16.0%	*17.4%*	***χ***^***2***^ ***= 12.8, p = 0.002,*** ***v = 0.43, BF***_***10***_ ***= 30.6***	***χ***^***2***^ ***= 9.4, p = 0.002,*** ***d = 1.03***, **BF**_**10**_ **= 34.2**	***χ***^***2***^ ***= 8.3, p = 0.004,*** ***d = 0.98***, **BF**_**10**_ **= 22.0**	*χ*^*2*^ = 0.1, *p* = 0.9, d = 0.09, BF_10_ = 0.3
Relationship status (% single)	60.0%	24.0%	*43.5%*	***χ***^***2***^ ***= 6.0, p = 0.049,*** ***v = 0.30, BF***_***10***_ ***= 2.1***	***χ***^***2***^ ***= 6.0, p = 0.01,*** ***d = 1.03***, **BF**_**10**_ **= 6.5**	*χ*^*2*^ = 1.2, *p* = 0.28, d = 0.34, BF_10_ = 0.6	*χ*^*2*^ = 2.0, *p* = 0.2, d = 0.42, BF_10_ = 0.9
Education level				***χ***^***2***^ ***= 19.4, p = 0.01,*** ***v = 0.38, BF***_***10***_ ***= 13.8***	***χ***^***2***^ ***= 12.3, p = 0.02,*** ***d = 1.23***, **BF**_**10**_ **= 23.4**	***χ***^***2***^ ***= 12.3, p = 0.02,*** ***d = 1.27***, **BF**_**10**_ **= 20.8**	*χ*^*2*^ = 5.8, *p* = 0.2, d = 0.74, BF_10_ = 0.2
No qualifications	25.0%	0.0%	*4.3%*				
GCSE/O’ levels	20.0%	16.0%	*0.0%*				
A’ levels	10.0%	8.0%	*13.0%*				
Vocational/College	25.0%	12.0%	*21.7%*				
University/Professional	20.0%	64.0%	*60.9%*				
Body mass index	31.1 ± 7.6	26.9 ± 6.7	24.5 ± 4.4	***F (62) = 5.7, p = 0.006,*** ***d = 0.84, BF***_***10***_ ***= 4.9***	Tukey HSD *p* = 0.10	***Tukey HSD p = 0.004***	Tukey HSD *p* = 0.40

Note: Bold + italics = significant *p*-value; d = Cohen's d; v = Cramer's v; BF = Bayes Factor.

Selection criteria excluded individuals with significant physical illness (e.g., cardiovascular or hepatic disease), Raynaud's phenomenon, substance use in the preceding 72 h and polydipsia (as this may confound accurate assessment of HPA parameters (Goldman et al., 2007)). Participants were classified as voice-hearers (both groups) if they reported at least weekly voices, as indicated by a score of ≥1 on the AVH frequency items of the Psychotic Symptoms Rating Scale (PSYRATS; Haddock et al., 1999). For clinical participants to be included, they had to be using secondary mental health services in relation to psychotic experiences. Participants were not recruited if they suffered from organic or drug-induced psychoses or were acutely unwell. NCVHs were recruited if they experienced AVHs for at least 5 years to avoid potentially prodromal individuals; if they had never sought help (either themselves, or someone on their behalf) from mental health services in relation to any psychotic experience; and had never received secondary care for any mental health issue, in line with recent research by Peters and colleagues (Peters et al., 2016). HCs were included if they had no current or past presence of auditory verbal hallucinations. Previous mental health complaints including anxiety and depression were accepted in both NCVHs and HCs unless they had necessitated secondary care intervention.

### 2.2 Materials

#### 2.2.1 Experimental measures

##### 2.2.1.1 Socially Evaluated Cold Pressor Test

The SECPT adapts the Cold Pressor classic paradigm, where a hand is exposed to cold, painful stimuli such as ice water in order to elicit a stress-response, by including a psychosocial component to exacerbate the HPA response (Schwabe et al., 2008). In the SECPT participants are informed that their facial expressions will be filmed during the task, they are asked to maintain eye contact with a camera set up in front of them, and an experimenter sits adjacent to them and pretends to take notes. Conventional freezer ice packs were used to cool down the water, and temperature was measured using a digital thermometer to ensure that all participants were exposed to temperatures between 0 and 3 °C. Maximum exposure was set at 3 min, after which participants were asked to remove their hand from the water if they had not already done so. Salivary and subjective stress measures were measured at two timepoints before exposure (−10 min and 0 min) and at 5 timepoints following exposure (0 min, +15 min, +30 min, +45 min and + 60 min). During the rest phase, all participants were presented with a 60-min nature documentary (BBC Natural History Unit, 2011) to provide a controlled stimulus and all SECPT sessions were held in the afternoon to account for diurnal HPA patterns.

##### 2.2.1.2 Saliva samples

Salivary cortisol and α-amylase levels were measured using Salivettes (Rommelsdorf, Germany). Freezing at −20 °C occurred within 2 h of collection for all SECPT samples. Participants were asked to gently chew the Salivettes for each collection, and were instructed not to touch samples with their hands. All samples were analysed at the ViaPath lab at King's College Hospital, using Salimetrics enzyme linked assays. Inter-assay coefficients of variations were 7.06 for cortisol and 6.26 for α-amylase.

#### 2.2.2 Questionnaire measures

##### 2.2.2.1 Psychotic Symptoms Rating Scale – AVH

The Psychotic Symptoms Rating Scale for Auditory Verbal Hallucinations (PSYRATS-AVHs) is a scale to assess dimensions of AVHs (Haddock et al., 1999). It is administered in a semi-structured interview where 11 items are scored on a 5-point Likert scale (0–4). A four factor structure comprising distress (negative content, distress and control), frequency (frequency, duration and disruption), attribution (location and beliefs about origin) and loudness was utilised (Woodward et al., 2014).A further item was included to assess age of onset of AVHs. The PSYRATS is a frequently used assessment tool that has repeatedly demonstrated validity and reliability in clinical and non-clinical populations (Daalman et al., 2011; Diederen et al., 2012; Haddock et al., 1999; Sommer et al., 2010; Woodward et al., 2014).

##### 2.2.2.2 Stress appraisal measure

To measure subjective anticipatory stress-appraisal several subscales (Threat; Centrality; Uncontrollability) of the Stress Appraisal Measure (SAM; Peacock and Wong, 1990)) were used, combining how threatening, central (i.e., perceived as important to oneself) and uncontrollable the task was anticipated to be. The 12 selected items were scored on a 5-point Likert scale from 1 to 5, with total scores ranging from 5 to 60, and higher scores indicating greater anticipatory stress-appraisal. The subscales have shown good validity (Peacock and Wong, 1990). The 12 item scale utilised here was found to be reliable in the present study (Cronbach's α = 0.90).

##### 2.2.2.3 Visual analogue scales

A 9-item visual analogue scale (VAS) was created to assess subjective stress-reactivity, with total scores ranging from 0 to 16 (one point for each centimetre interval) and higher scores indicating greater subjective stress levels. Participants were asked how stressed, in pain, anxious, angry, relaxed (reverse coded), threatened, embarrassed, socially judged and expecting of positive vs negative consequences they were, to reflect an array of possible stress responses to the SECPT. A Principal Component Analysis was carried out to determine whether the scale represented a latent factor indicative of a general subjective stress reaction, using VAS scores at baseline. The Kaiser-Meyer-Olkin measure verified sampling adequacy (KMO = 0.82) and Bartlett's test of sphericity was significant (χ^2^ (36) = 289.2, p 〈001). A one factor structure was most appropriate (Eigenvalue = 4.44). It was decided to suppress items with coefficients below 0.5 (Field, 2014), leading to exclusion of one item (expecting positive vs negative consequences), with the lowest factor loading being 0.53. Cronbach's α indicated the scale to be reliable (0.85).

##### 2.2.2.4 Demographics and covariates

A demographics questionnaire was created to assess age, gender, ethnicity, living status, relationship status, employment status, level of achieved education and native language of participants. To investigate potential confounding variables that may lead to variation in salivary biomarker data, a questionnaire was created to control for biological factors that may impact on physiological stress-function. Items assessed included age (Holochwost et al., 2017; Skoluda et al., 2017), gender(Kirschbaum et al., 1999), BMI (Skoluda et al., 2017), current medications, current medical diagnoses, time of last meal, time of last drink, time of last cigarette (if applicable; Skoluda et al., 2017)), menstrual phase (Kirschbaum et al., 1999), strenuous exercise in the preceding 72 h (Bonato et al., 2017), and stress exposure in the preceding 24 h (Skoluda et al., 2017).

### 2.3 Procedure

#### 2.3.1 Recruitment

For all potential participants, capacity to consent was assessed following standard procedures to determine capacity (Owen et al., 2013), informed consent was obtained and eligibility was assessed using inclusion/exclusion criteria. This protocol was carried out as part of a larger study reported on elsewhere (Baumeister et al., Under Review).

All participants were screened by a study doctor before completing the Socially Evaluated Cold Pressor Task (SECPT). At baseline of the SECPT, a saliva sample, subjective stress (VAS) as well as threat appraisal (SAM) measures were taken. Then, 10 min after the baseline time point, participants completed a second VAS and saliva sample, and were asked to immerse their hand up to and including the wrist in ice water, and keep their hand submersed for as long as possible. Participants who still had their hand submersed after 3 min were asked to remove it at that point. A record of the time their hand was submersed was taken for each participant. Upon completion, the third saliva sample and VAS score were obtained. Over the remaining 60 min, saliva and subjective stress (VAS) were measured every 15 min. Participants then completed the questionnaire battery. Following their participation, participants were fully debriefed.

### 2.4 Statistical analyses

#### 2.4.1 Questionnaire measures

Statistical analyses were carried out using SPSS for Windows (IBM Corp, 2015), and JASP (JASP Team, 2016). *P*-values below the 0.05 two-tailed threshold were accepted as statistically significant. In instances of post-hoc tests, False Discovery Rate (FDR)-adjusted *p*-values are reported to control for type II error rate. Bayes factors of 3 and above were interpreted as sufficient evidence for the alternative hypothesis, and Bayes factors of 1/3 and below as sufficient evidence for the null hypothesis (Kass and Raftery, 1995). Bayesian factors were calculated to provide an additional metric for evaluation of findings, expressing the likelihood of data supporting the hypotheses. Normal distribution of data was checked by Q-Q plots and Kolmogorov-Smirnov tests, and Levene's test was used to assess homogeneity of variance. For group comparisons of sociodemographic and questionnaire data, separate analyses were carried out for each questionnaire variable (dependent variables (DVs)); with group (i.e., CVH vs NCVH vs HC) as the independent variable (IV). Chi-square analyses were carried out for binary DVs. For normally distributed data, independent samples *t*-tests (CVH vs NCVH) and ANOVA (CVH vs NCVH vs HC) were used for group comparisons on continuous variables. In group comparisons for non-normally distributed continuous variables, non-parametric Mann-Whitney U (CVH vs NCVH) tests were used.

For all cortisol and α-amylase analyses, associations of DVs with potential covariates were assessed which were included if significant. To establish normal distribution of cortisol and α-amylase data, logarithmic transformations (log_n_) were undertaken for all analyses.

For assessment of salivary cortisol and α-amylase response to the SECPT, repeated-measures ANCOVAs were carried out with group as the between-participants IV, timepoint as the within participants IV and log-transformed cortisol and α-amylase as the DV. For cortisol, bivariate correlations revealed current progesterone treatment as a potential covariate. For α-amylase, menstrual phase was identified as a potential covariate. Post-hoc testing of group-specific outcomes was carried out via pairwise comparisons as well as lower order ANOVAs for specific timepoints. For assessments of peak cortisol and α-amylase responses between groups, repeated-measures ANCOVAs for cortisol and α-amylase (with the covariates identified above) were carried out from post-SECPT to 15 min for cortisol, and pre-SECPT to post-SECPT for α-amylase. For analysis of subjective stress levels during the SECPT, a repeated-measures mixed ANOVA was carried out on total VAS scores (DV) across timepoints (within-participant IV) and group as the between-participants IV. For recovery of subjective stress-levels, paired-samples *t*-tests within groups were carried out on VAS scores from post-SECPT to 15 min.

## 3 Results

### 3.1 Sample characteristics

The present study comprised 20 CVHs, 23 NCVHs and 23 HCs. Participant groups did not differ significantly on age, ethnicity, and first language. However, group differences were found for gender, employment status, living status, relationship status, education level and body-mass index (Table 1). Clinical characteristics of CVHs are presented in Table 2. Comparing AVH parameters between CVHs and NCVHs (Table 3), CVHs had significantly higher scores on Frequency, Loudness and Distress subscales of the PSYRATS, but did not differ on the attribution subscale. NCVHs had a significantly earlier age of onset of AVHs than CVHs.

**Table 2:** Clinical characteristics CVHs (n, %).

Diagnosis	
Schizophrenia	11, 55%
Schizoaffective disorder	1, 5%
Psychosis NOS	3, 15%
Other affective disorders	5, 25%


Psychiatric medications	
Typical antipsychotics	0, 0%
Atypical antipsychotics	11, 55%
Clozapine	6, 30%
No antipsychotics	3, 15%
Antidepressants	9, 45%
Mood stabilisers	3, 15%

**Table 3:** AVH Results (Mean ± SD unless specified otherwise).

	CVHs (n = 20)	HVHs (*n* = 23)	Statistics
PSYRATS			
Frequency	7.0 ± 2.0	3.3 ± 1.1	***U = 15, Z = 5.5, p < 0.001, d = 2.67***, BF_10_ = 4.0 × 10^7^
Loudness	2.1 ± 0.9	1.4 ± 0.8	***U = 138, Z = 2.8, p = 0.005, d = 0.83***, BF_10_ = 4.4
Attribution	5.3 ± 1.4	5.7 ± 1.1	*U = 407, Z = 1.4, p = 0.20, d = 1.27*, BF_10_ = 0.5
Distress	16.2 ± 3.8	1.5 ± 2.0	***U = 1, Z = 5.7, p < 0.001, d = 3.20***, BF_10_ = 7.3 × 10^16^
Age of Onset:	27.3 ± 11.4	15.3 ± 11.2	***t (43) = 3.5, p = 0.001***, BF_10_ = 30.4

Note: bold + italics = significant *p*-value; d = Cohen's d; BF = Bayes Factor.

### 3.2 Socially Evaluative Cold Pressor Test

One-way ANOVA showed that there were no significant differences in water temperature between groups (F (2, 63) = 0.8, *p* = 0.46, d = 0.31, BF_10_ = 0.2), nor did participant groups differ in how long they kept their hand in the ice water (F (2, 63) = 1.1, *p* = 0.35, d = 0.37), confirming equal exposure across groups.

#### 3.2.1 Subjective response

One-way ANOVA showed significant group differences in anticipatory SAM scores prior to the task across groups (F(2,63) = 7.6, *p* = 0.001, d = 0.39, BF_10_ = 29.4). As predicted, post-hoc comparison showed that this was due to significantly higher SAM scores in CVHs (M = 23.2, SD = 1.6) compared to both NCVHs (M = 17.1, SD = 1.5, *p* = 0.009) and HCs (M = 15.1, SD = 1.5, *p* = 0.003), who did not differ from each other (*p* = 0.35).

**Fig. 1:**
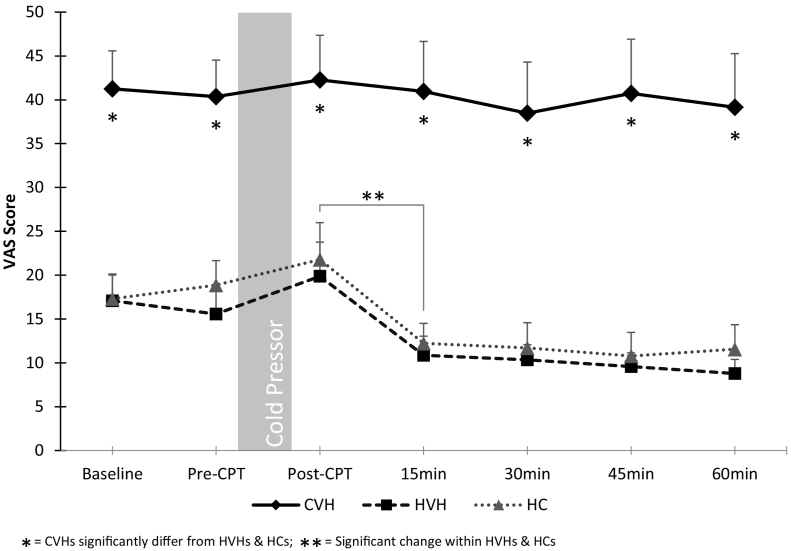
VAS scores by group by timepoint (mean ± SE).

Repeated measures ANOVA revealed significant effects of timepoint (F (1.9, 118.1) = 4.8, *p* = 0.01, d = 0.14, BF_10_ = 292.4) and group (F (2, 63) = 31.7, *p* < 0.001, d = 1.00, BF_10_ = 3.2 × 10^7^) on VAS scores. Post-hoc group comparison showed significantly higher VAS scores in CVHs compared to both NCVHs (*p* = 0.002) and HCs (p = 0.002), but no differences between NCVHs and HCs (*p* = 0.66), as predicted (Fig. 1). There was no significant interaction between timepoint and group (F (3.8, 118.1) = 0.62, *p* = 0.64, d = 0.04, BF_M_ = 0.03.

For recovery post-SECPT, NCVHs (t(24) = 2.5, *p* = 0.03, d = 0.59, BF_10_ = 2.3) and HCs (t(22) = 3.0, *p* = 0.02, d = 0.46, BF_10_ = 7.1) showed a significant decrease in VAS scores from Post-SECPT to 15 min, which was not observed in CVHs (t(19) = 0.2, *p* = 0.86, d = 0.06, BF_10_ = 0.2).

#### 3.2.2 Cortisol response

**Fig. 2:**
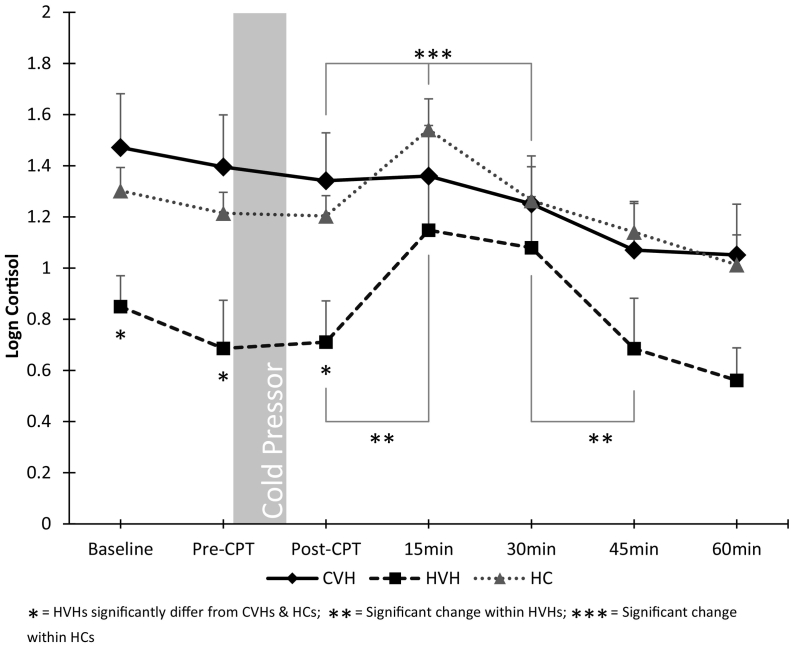
Log_n_ cortisol values by group by timepoint (mean ± SE).

Repeated measures ANCOVA, controlling for progesterone treatment, revealed a significant effect of timepoint on cortisol levels (F (3.1, 191.8) = 15.9, *p* < 0.001, d = 0.41, BF_10_ = 4.0 × 10_12_), as well as a significant interaction with group (F (6.3, 191.8) = 2.8, *p* = 0.01, d = 0.17, BF_M_ = 8.4; Fig. 2). Test of between-participants effects showed trend-level significance (F (2, 61) = 2.88, *p* = 0.064, d = 0.61, BF_10_ = 1.6). Pairwise comparison showed that, as predicted, CVHs had trend-level higher cortisol levels than NCVHs (*p* = 0.07), however, contrary to predictions they did not differ from HCs (*p* = 0.97). Unexpectedly, NCVHs also showed trend-level lower cortisol levels than HCs (p = 0.07). Lower order ANOVAs revealed significant group differences at Baseline (F(2, 63) = 5.0, *p* = 0.02, d = 0.28, BF_10_ = 5.2; CVH vs NCVH: *p* = 0.01, CVHs vs HC: *p* = 0.50, NCVH vs HC: *p* = 0.04); Pre-SECPT (F(2, 63) = 5.1, p = 0.02, d = 0.28, BF_10_ = 5.3; CVH vs NCVH: p = 0.01, CVHs vs HC: p = 0.50, NCVH vs HC: p = 0.04), and 0 min Post-SECPT (F(2, 63) = 5.1, p = 0.02, d = 0.28, BF_10_ = 5.5; CVH vs NCVH: p = 0.01, CVHs vs HC: *p* = 0.51, NCVH vs HC: p = 0.04), with lower cortisol in NCVHs compared to both CVHs and HCs, who did not differ from each other.

To assess blunting of the peak cortisol response (i.e., from pre-SECPT to 15 min post-SECPT), repeated measures ANCOVA controlling for progesterone revealed a significant effect for timepoint on cortisol levels (F (1, 62) = 19.0, *p* < 0.001, d = 1.11, BF_10_ = 343.1), with a trend-effect effect of group on cortisol (F (2, 62) = 2.7, *p* = 0.08, d = 0.59, BF_10_ = 1.4), and a significant interaction effect of group and timepoint on cortisol (F (2, 62) = 4.2, *p* = 0.02, d = 0.74, BF_M_ = 5.2). Post-hoc paired-samples *t*-test within groups revealed that, as predicted, there was no significant change in cortisol levels in CVHs (t (19) = 0.5, *p* = 0.61, d = 0.03, BF_10_ = 0.2), whilst cortisol levels increased significantly in NCVHs (t (22) = 3.0, *p* = 0.009, d = 0.48, BF_10_ = 9.0) and HCs (t(22) = 3.6, *p* = 0.006, d = 0.63, BF_10_ = 29.5).

#### 3.2.3 α-Amylase response

Repeated measures ANCOVA, controlling for menstrual cycle, also suggested a violation of Mauchly's test of sphericity for α-amylase, and therefore the Greenhouse-Geisser statistic was used. This revealed no significant effects for timepoint on α-amylase levels (F (4.0, 244.4) = 0.7, *p* = 0.60, d = 0.24, BF_10_ = 0.02), nor was there an interaction with group (F (8.0, 244.4) = 0.8, *p* = 0.63, d = 0.19, BF_M_ = 4.0 × 10^−4^). Test of between-participants effects showed no significant effect of group (F (2, 61) = 0.1, *p* = 0.87, d = 0.01, BF_10_ = 0.4). Similarly, repeated measures ANCOVA investigating the peak α-amylase response revealed no significant effect for timepoint on α-amylase levels (F (1, 61) = 1.2, *p* = 0.27, d = 1.00, BF_10_ = 0.5), no effect of group (F (2, 61) = 0.2, *p* = 0.79, d = 0.18, BF_10_ = 0.6), and no interaction effect of group and timepoint (F (2, 61) = 0.97, *p* = 0.38, d = 0.36, BF_M_ = 0.1).

## 4 Discussion

### 4.1 Findings

As hypothesised, subjective stress-response to the psychophysiological stressor differed significantly between groups, with higher anticipatory stress appraisals and subjective stress levels in CVHs. Furthermore, they appeared to show no subjective recovery following the SECPT, whereas subjective stress levels in NCVHs and HCs decreased following exposure. CVHs also showed similar overall cortisol levels to HCs during the SECPT yet failed to show the increase in cortisol secretion in response to the stressor, as was observed in NCVHs and HCs. This blunted stress response is in line with our hypotheses, as well as previous evidence in psychosis (Ciufolini et al., 2014; Rubio et al., 2015; Pruessner et al., 2017). However, the hypothesis of exacerbated α-amylase response in CVHs was not supported. To the best of our knowledge, salivary α-amylase responses to psychosocial stressors have not been previously assessed in psychosis patients but there is recent evidence that the SECPT fails to elicit α-amylase responses in healthy controls (Becker et al., 2019), consistent with our findings in controls, suggesting either that salivary α-amylase may not be sensitive to effects of the SECPT or the SECPT does not induce marked effects on the autonomic nervous system balance. We believe that this likely underlies the divergence from the literature on altered α-amylase in schizophrenia, where levels were shown to be higher in clinical participants but were either assessed unstimulated (Ieda et al., 2014; Inagaki et al., 2010) or in response to awakening (Monteleone et al., 2015).

Intriguingly, NCVHs showed trend-level lower total cortisol levels during the SECPT than both HCs and CVHs. There are several studies reporting that childhood (Carpenter et al., 2011, Carpenter et al., 2007; Voellmin et al., 2015) as well as lifetime adverse events in healthy individuals (Elzinga et al., 2008; Lovallo et al., 2012) are associated with similar HPA stress-reactivity patterns (i.e., reduced cortisol levels in stress paradigms) to those observed in the NCVHs in this study, and NCVHs have been found to report greater levels of trauma exposure than HCs (Baumeister et al., 2020, Baumeister et al., 2017). However, it remains unclear whether the decreased HPA activity of trauma-exposed healthy individuals in psychosocial stress paradigms is in fact indicative of resilience (Voellmin et al., 2015). Of note, a recent investigation of adversity exposure patterns in 57 CVHs and 45 NVCHs found that whilst exposure to sexual and non-sexual victimisation, discrimination and socio-economic disadvantage did not differ between groups, there was a significant difference in family history of psychosis, as well as cannabis and other substance use (Baumeister et al., 2020). Cannabis use is associated with exacerbated HPA activity in youths at high risk for psychosis (Carol et al., 2017), suggesting that a potential source of divergence in the HPA trajectories could be related to dispositional factors as well as maladaptive interaction effects with cannabis use in adolescence.

## 5 Strengths and limitations

This is the first study to characterise HPA-alterations specifically in voice-hearers with and without a need for care, thus allowing for specific evaluation of the role of stress-reactivity in need for care. Detailed assessments of psychophysiological stress-reactivity were undertaken, and the study employed a symptom-focused design, which is preferable to diagnosis-based designs for the identification of mechanisms underlying psychotic phenomena (Insel et al., 2010). There is well documented evidence for lower pain sensitivity in schizophrenia patients (Stubbs et al., 2015), which could potentially impact the stress response during the SECPT. However, analyses showed no significant differences in the time CVHs could endure the ice water, suggesting that differential pain sensitivity did not affect the results.

Several limitations need to be highlighted. A confounding effect of gender across voice-hearer groups cannot be ruled out. Further, all CVHs in the present study were medicated. Antipsychotic medications may particularly impact on HPA-reactivity in that they appear to attenuate the overall activity of the HPA axis (Pruessner et al., 2017). Finally, a cut-off of at least weekly voice-hearing was employed to ensure sufficient AVH frequency and comparability in both CVHs and NCVHs. That may however have biased our clinical sample toward a treatment resistant group.

## 6 Future directions

Several important clinical and research implications can be drawn from the present study. First, the present data suggest that stress-function differs depending on the need for care of voice-hearers. Future longitudinal research is needed to assess whether the type of stress response found in the NCVHs is a marker of resilience. In this study, the evidence suggests that stress function is differentially related to AVHs, depending on clinical status. Capturing individuals before or the around the onset of AVHs and assessing parameters of stress-function as well as wider psychopathology may help elaborate to what degree the divergence of stress-function in CVHs and NCVHs is related to the onset of AVHs. The lower cortisol levels of NCVHs during the SECPT could be related to wider stress-resilience of the HPA axis, which could explain their non-clinical status. Further, to which degree a change in need for care, i.e., therapeutic response to psychological or pharmacological treatments, is also associated with normalisation of stress-function should be evaluated.

Finally, the association of specific HPA-activity patterns with other biomarkers implicated in need for care needs to be investigated. The dopamine hypothesis proposes that elevated dopaminergic activity in the striatum underlies aberrant salience and the formation of delusional explanations and beliefs (Howes and Kapur, 2009; Kambeitz et al., 2014). NCVHs do not present with the upregulated dopamine synthesis capacity seen in psychosis patients (Howes et al., 2013). The mesolimbic dopamine system is highly responsive to glucocorticoid secretion (Marinelli and Piazza, 2002), and several studies have demonstrated that acute psychosocial stress leads to increased regional dopamine signalling (Mizrahi et al., 2014; Nagano-Saito et al., 2013; Pruessner et al., 2004; Vaessen et al., 2015). Investigating the stress-dopamine link in clinical and NCVHs may further explain pathophysiological adaptations associated with need for care, and the link between AVHs and delusional ideation.

## 7 Conclusions

The present study demonstrates significant alterations to the stress-reactivity of clinical and NCVHs, with significant differences depending on need for care status. HPA-reactivity is specifically implicated in the pathophysiology of CVHs. Contrary to our expectations, NCVHs showed HPA-patterns divergent from HCs, raising the interesting possibility of specific resilience markers. Activity of the ANS did not differ between groups in the present study.

## Contributors

DB carried out the study preparation, data collection, analyses and write-up. EP and OH oversaw the project as academic supervisors, contributed to study design and manuscript preparation. TP aided with recruitment and medical screenings.

## Data statements

An anonymised version of the data set is available from the authors upon request.

## Declaration of competing interest

The authors declare no conflicts of interest.
